# Epigenetic regulation of functional candidate genes for milk production traits in dairy sheep subjected to protein restriction in the prepubertal stage

**DOI:** 10.1186/s12864-023-09611-y

**Published:** 2023-09-01

**Authors:** P. A. S. Fonseca, A. Suárez-Vega, C. Esteban-Blanco, R. Pelayo, H. Marina, B. Gutiérrez-Gil, J. J. Arranz

**Affiliations:** Departamento de Producción Animal, Facultad de Veterinaria, Universidad de León, Campus de Vegazana S/N, 24071 León, Spain

**Keywords:** Differentially methylated regions, Mammary gland, Protein restriction, Dairy, Sheep

## Abstract

**Background:**

As the prepubertal stage is a crucial point for the proper development of the mammary gland and milk production, this study aims to evaluate how protein restriction at this stage can affect methylation marks in milk somatic cells. Here, 28 Assaf ewes were subjected to 42.3% nutritional protein restriction (14 animals, NPR) or fed standard diets (14 animals, C) during the prepubertal stage. During the second lactation, the milk somatic cells of these ewes were sampled, and the extracted DNA was subjected to whole-genome bisulfite sequencing.

**Results:**

A total of 1154 differentially methylated regions (DMRs) were identified between the NPR and C groups. Indeed, the results of functional enrichment analyses of the genes harboring these DMRs suggested their relevant effects on the development of the mammary gland and lipid metabolism in sheep. The additional analysis of the correlations of the mean methylation levels within these DMRs with fat, protein, and dry extract percentages in the milk and milk somatic cell counts suggested associations between several DMRs and milk production traits. However, there were no phenotypic differences in these traits between the NPR and C groups.

**Conclusion:**

In light of the above, the results obtained in the current study might suggest potential candidate genes for the regulation of milk production traits in the sheep mammary gland. Further studies focusing on elucidating the genetic mechanisms affected by the identified DMRs may help to better understand the biological mechanisms modified in the mammary gland of dairy sheep as a response to nutritional challenges and their potential effects on milk production.

**Supplementary Information:**

The online version contains supplementary material available at 10.1186/s12864-023-09611-y.

## Introduction

The world population is constantly increasing, and the population size is expected to reach 10.9 billion by 2100 [1]. Consequently, a demand for increased production of food with high nutritional value is expected to emerge. However, sustainability and animal welfare standards must be maintained with this increase in production, which is a challenge for producers [2]. In animal production systems, management decisions related to feeding strategies can account for up to 75% of all variable costs in a herd, and protein accounts for a high proportion of these costs [3–5]. Several additional issues are associated with protein intake in livestock species. For example, in Europe, a disturbance of the nutrient cycle related to the supply of protein for animal feed is observed due to geographical separation, mainly concerning the import of soybeans from subtropical regions [6]. Consequently, the protein intake in flocks must be reviewed as a crucial component in controlling the sustainability of the whole production chain. Equally important, protein prices and availability in the market have recently been highly volatile. Therefore, feeding strategies in livestock herds are under constant pressure to change and adapt.

In general, nutrition is a major component affecting sheep milk composition, and appropriate nutritional management is directly related to improvements in the nutritional value of the milk [7]. In ruminants, postnatal mammary growth occurs at an allometric rate before puberty and returns to an isometric rate after puberty [8]. In general, sheep reach puberty at 6–8 months old, and there is a consensus that the ovine mammary allometric growth occurs between 3 and 4 months of age [9, 10]. It is well documented that elevated nutrient intake during this allometric growth phase results in reduced parenchymal mass and DNA [11]. The amplification of genomic DNA during lactation is hypothesized to be associated with increasing gene copies to support a high rate of RNA and protein synthesis [12]. Therefore, the nutrient supply during the critical developmental window and the diet composition are important factors affecting proper animal development and production to obtain high-quality products that meet consumer demands. Generally, farmers avoid reducing nutrition costs during lactation, trying to maintain the ewe’s productive potential. On the other hand, the effects of restricted feeding (reduction in concentrate) during the prepubertal stage of Dorset ewes were shown to improve mammary gland development without affecting growth performance [13]. In addition, in dairy cows, supplementation with protein during the prepubertal stage did not seem to affect milk production later in the lives of these animals [14, 15]. Interestingly, a previous study by our group revealed the absence of an effect of a 42.3% nutritional protein restriction (NPR) in the prepubertal growth stage on economically important traits in Spanish Assaf ewe lambs, such as milk somatic cell counts (SCC) [16]. This reinforces the notion that reducing protein intake in the diet of replacement ewe lambs may be performed without negatively impacting their milk production as adult ewes. The absence of phenotypic differences between groups of animals fed under regimes with substantial differences in protein intake during prepuberty might be due to compensatory biological mechanisms. Indeed, among the potential biological alterations associated with NPR, DNA methylation stand out as relevant candidates due to previously reported effects caused by protein restriction [17, 18]. Modifications in nutritional status can directly affect enzymes related to epigenetic processes or even change the availability of substrates for those enzymes [19]. Consequently, these alterations might affect the later expression of genes related to alterations in the mammary gland of ewes subjected to NPR in the prepubertal stage.

In light of the above, the main objectives of this study were 1) to evaluate the impact on the genome methylation state of the milk somatic cells during the second lactation of Assaf ewes subjected to NPR during the prepubertal stage and 2) to identify DNA methylation in functional candidate genes that are associated with regulatory mechanisms related to milk production traits in dairy sheep.

## Material and methods

### Sampling and nutritional challenge

Initially, 40 lamb ewes from a single flock in the northwest region of Castilla y León (Spain) that were transported to the facilities of the IGM in León were fed ad libitum with a standard diet for replacement ewe lambs providing 16% crude protein until three months of age and were subsequently divided into two groups. The two experimental groups were composed of 20 NPR and 20 C animals. To evaluate the impact of the protein restriction challenge due to a trade market problem and a shortage of concentrate inputs, the C ewes received the standard diet mentioned above for 64 d; during the same period, the NPR ewes received the same diet but replacing soybean meal with maize and barley grains. The diet composition offered to each group is shown in Supplementary Table 1. The NPR diet was planned to produce an intense reduction in dietary crude protein (42.3%) but has other notable qualitative changes, such as a 16% reduction in acid detergent fiber (ADF), a nearly 29% increase in acid detergent lignin (ADL) and ether extract. The diets were offered ad libitum for both groups. The 64-d NPR period in the prepubertal stage was coincident with the allometric growth of the mammary gland [11]. After the experimental period, the animals were fed ad libitum with the standard diet for replacement ewe lambs till milk sampling. The ewes passed through two gestational and lactation periods, where in the second lactation, the milk samples were collected for DNA extraction. Only 28 (out of the initial 40 challenged ewes) were pregnant in the second lactation. Therefore, these ewes constituted the sample used for the methylation marks analysis.

### DNA extraction and whole-genome bisulfite sequencing

The milk samples (100 mL, 50 from each teat) obtained from 28 animals out of the 40 initially used in the nutritional challenge were collected once before the morning milking. Milk somatic cells were obtained using the following protocol: 1) addition of 50 µL of EDTA 0.5 mol/L to each sample; 2) centrifugation at 6000 × g for 10 min at 4 °C; 3) transfer of the pellet to a 2 µL microtube and centrifugation at 7000 g for 10 min at 4 °C; 4) removal of the supernatant and addition of 1 mL of wash buffer (15 mmol/L Tris–HCl (pH 7.4–7.6), 25 mmol/L NaCl, 5 mmol/L MgCl_2_, 15 mmol/L Na_2_HPO_4_, 2.5 mmol/L EDTA, 1% sucrose); 5) centrifugation at 3000 g for 3 min at 4 °C; 6) removal of the supernatant and dissolution of the pellet in 1 mL of wash buffer; 7) centrifugation at 3,000 g for 3 min at 4 °C (the last two steps can be repeated if the supernatant is not clear); finally, removal of the supernatant and DNA extraction. DNA extraction was performed using the MasterPure™ Complete DNA and RNA Purification Kit. The SCC and the DNA concentration (ng/mL) obtained for each sample are available on Supplementary Fig. 1. The samples were used for paired-end (150 bp) library construction on the Novogene platform (Milton, Cambridge, United Kingdom), and libraries were sequenced on an Illumina NovaSeq 6000 system, with a minimum coverage depth of 20X for each sample. Detailed information regarding library preparation and whole genome bisulfite sequencing (WGBS) is available from Fonseca et al. (2022) [20]. The raw datasets derived from sequencing are available in the European Nucleotide Archive (ENA) repository under accession number PRJEB56589 (https://www.ebi.ac.uk/ena/browser/view/PRJEB56589).

### Differential methylation between nutritionally challenged and control sheep

The quality control of the 28 raw samples obtained from the aforementioned milk samples was performed using FastQC [21]. Subsequently, the reads were trimmed based on quality scores, adapters were removed, and short reads were filtered using the default options of Trim Galore software (version 0.6.5) [22]. Initially, the ovine reference genome, Oar_Ram_v2.0, was indexed by BowTie2 [23]. Subsequently, the Python script bs_seeker2-align.py from Bsseeker2 [24] was used to align the trimmed reads against the reference genome with the default options. The alignment output files were sorted by position using SAMtools software (version 1.15.1) [25], and duplicate reads were removed using Picard software (version 2.25) (https://broadinstitute.github.io/picard/). Finally, the methylation calling procedure was performed using the Python script bs_seeker2-call_methylation.py from Bsseeker2 using the default options. Additionally, methylated sites with fewer than ten reads mapped within the region were filtered out.

Differentially methylated loci (DMLs) and differentially methylated regions (DMRs) were identified using the R package DSS [26]. First, a simple average algorithm for smoothing was used to estimate mean methylation levels. The DMLs were identified by comparing the mean methylation levels in the NPR and C groups for each methylated site. The DMRs were identified based on regions harboring statistically significant methylated sites based on the following criteria: FDR-adjusted *P* value < 10^–5^ for methylated sites, minimum length of 50 bp, minimum number of 50 methylated sites, significant percentage of methylated sites being in the region (0.5), and a methylation difference greater than 0.1. Additionally, the DMRs mapped within regions located less than 50 bp from each other were merged into a single DMR. The DMRs were assigned to candidate genes and annotated for genomic context (promoter, intron, exon, and intergenic) with the R package genomation [27] using the gene annotation file from the ovine reference genome Oar_Ram_v2.0 (annotation release 104). For DMRs that were not mapped within a gene coordinate (intergenic regions), the closest gene was assigned to the DMR.

### Gene Ontology and metabolic pathway enrichment analysis and quantitative trait locus annotation

The R package gprofiler2 was used for GO term and KEGG [28, 29] and Reactome pathway enrichment analysis. Enriched status was defined based on a false discovery rate (FDR) < 0.05. Only GO terms with more than 2 and fewer than 1000 assigned genes were considered for enrichment status. A redundancy reduction analysis was performed using the rutils package in R. The go_reduce() function was used to estimate semantic similarities between the GO terms via the Wang method, where a graph-based strategy for computing semantic similarity using the topology of the GO graph structure is applied. Subsequently, terms with a similarity threshold higher than 0.7 were grouped. The smallest adjusted *P* value was assigned for the different GO terms grouped under the same parental GO term, and duplicates were removed. The enrichment analysis was performed individually for DMRs with higher methylation means in each group (NPR and C). Additionally, the GALLO R package [30] was used to annotate the colocalization of the genes harboring DMRs with quantitative trait loci (QTL) using SheepQTLdb from Animal QTLdb [31]. An interval of 250 kb downstream and upstream (500 kb in total) from the start and end coordinates of each DMR was considered for QTL annotation. The gff file from SheepQTLdb (Oar_Ram_v2.0) was edited to remove QTLs with lengths greater than 1 Mb. This approach was chosen to avoid the annotation of QTLs covering large regions of chromosomes, usually identified by linkage methods and using low-density microsatellite marker maps.

### Statistical analyses

The milk composition of the 28 ewes subjected to WGBS was recorded at 11 time points. The sampling points, represented as the mean (± standard deviation) days in milk were: 26.39 (± 8.02), 32.39 (± 8.02), 40.39 (± 8.02), 46.39 (± 8.02), 52.39 (± 8.02), 53.39 (± 8.02), 57.39 (± 8.02), 58.39 (± 8.02), 59.39 (± 8.02), 65.39 (± 8.02), 66.39 (± 8.02).

In all these points, the fat percentage (FP), protein percentage (PP), dry extract percentage (DE), SCC, and milk yield (MY) were assessed. Milk yield was measured by weighing the total milk produced by each animal during morning and evening milking. Milk FP, PP, and DE were determined by infrared spectrophotometry (ISO 9622:1999) using a MilkoScan FT6000 (Foss), combined with a fluoro-opto-electronic counter (Fossomatic 5000, Foss) for SCC (ISO 13366–2:2006). The average values of FP, PP, DE, and SCC were log-transformed and compared between the NPR and C groups using a generalized linear model, where the number of lambs born during the current lactation was included as a fixed effect. The MY values were maintained in the original scale and compared between NPR and C using the same model. The statistical analyses were performed using the statistical software R (R version 4.2.0) [32].

The Pearson correlation between the raw values of FP, PP, DE, SCC, and MY and the mean methylation level of each DMR was estimated using the *cor.test* function in R. The correlations were estimated for each milk trait (FP, PP, DE, SCC, and MY) individually for the samples of the NPR and C groups. Significant correlations were defined using the threshold of a *P* value < 0.05.

## Results

### Whole-genome bisulfite sequencing statistics

The mapping statistics for the reads obtained via WGBS are shown in Supplementary Table 2. An average mapping rate of 66.40 ± 3.32% was obtained, with values ranging from 59.70% to 69.10%. In eukaryotes, methylation can occur in three different contexts: CG, CHG, and CHH, where H is adenine (A), cytosine (C), or thymine (T). The mean percentages (± standard deviation) of methylated sites in CG, CHG and CHH contexts were 71.33 ± 1.15%, 1.34 ± 0.02%, and 1.42 ± 0.02%, respectively, in the NPR group. The means for the CG, CHG and CHH contexts for the control (C) group were 71.56 ± 0.97%, 1.34 ± 0.02%, and 1.41 ± 0.02%, respectively. Therefore, similar methylation patterns were observed in the NPR and C groups.

### Identification of DMLs and DMRs between NPR and control ewes

**Fig. 1:**
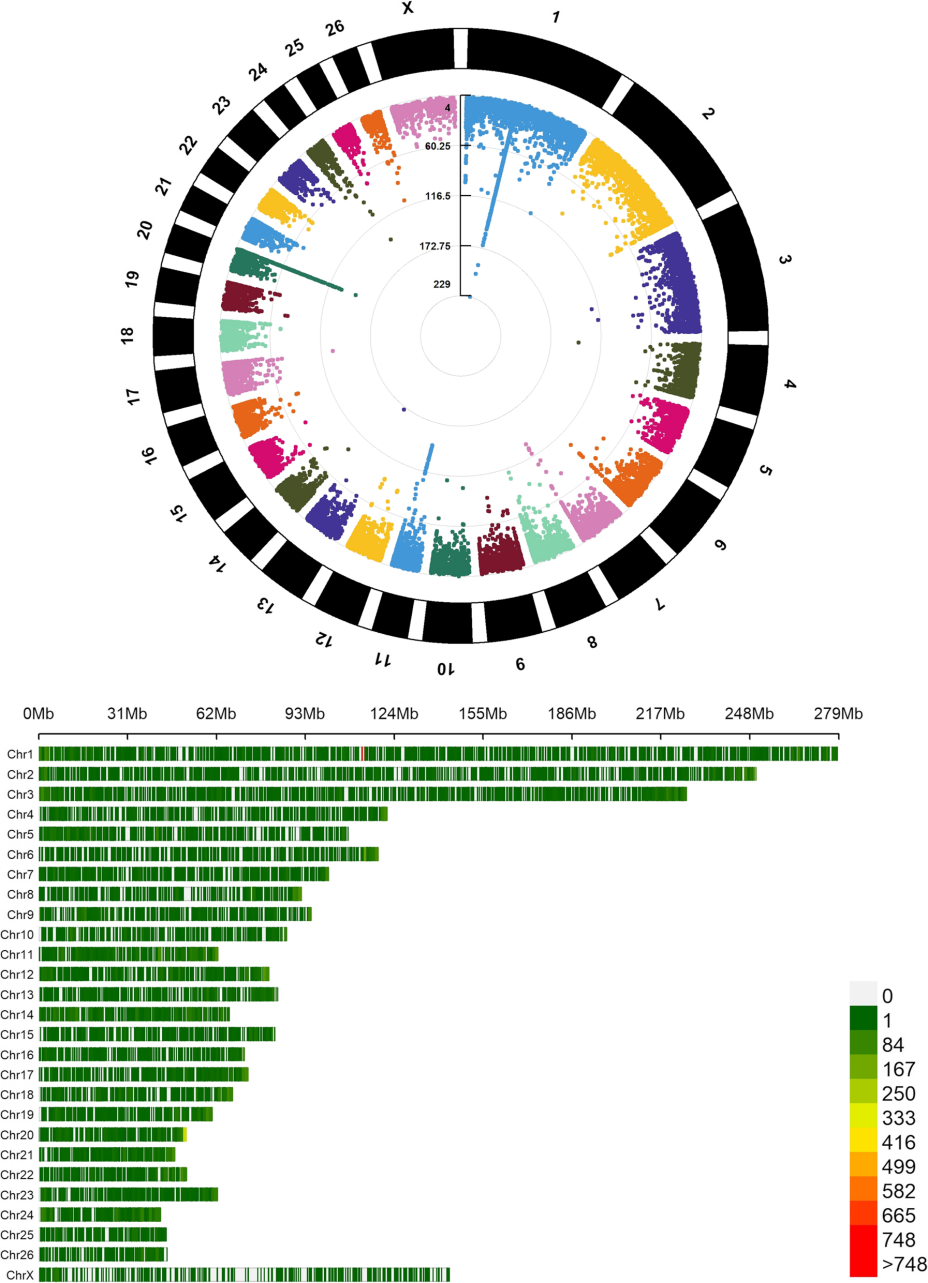
Circular Manhattan plot and genomic density distribution of differentially methylated loci (DMLs) identified in the comparison between the nutritional protein restriction (NPR) and Control group. In the circular Manhattan plot, the associated values for each DML is in a -log_10_ scale. In the density plot, the darker the red shade on the bar plots, the highest the density of the DMLs within the 1 Mb windows comprising the DMLs. The legend of the density plot represents the number of DMLs associated with each color scale

**Fig. 2:**
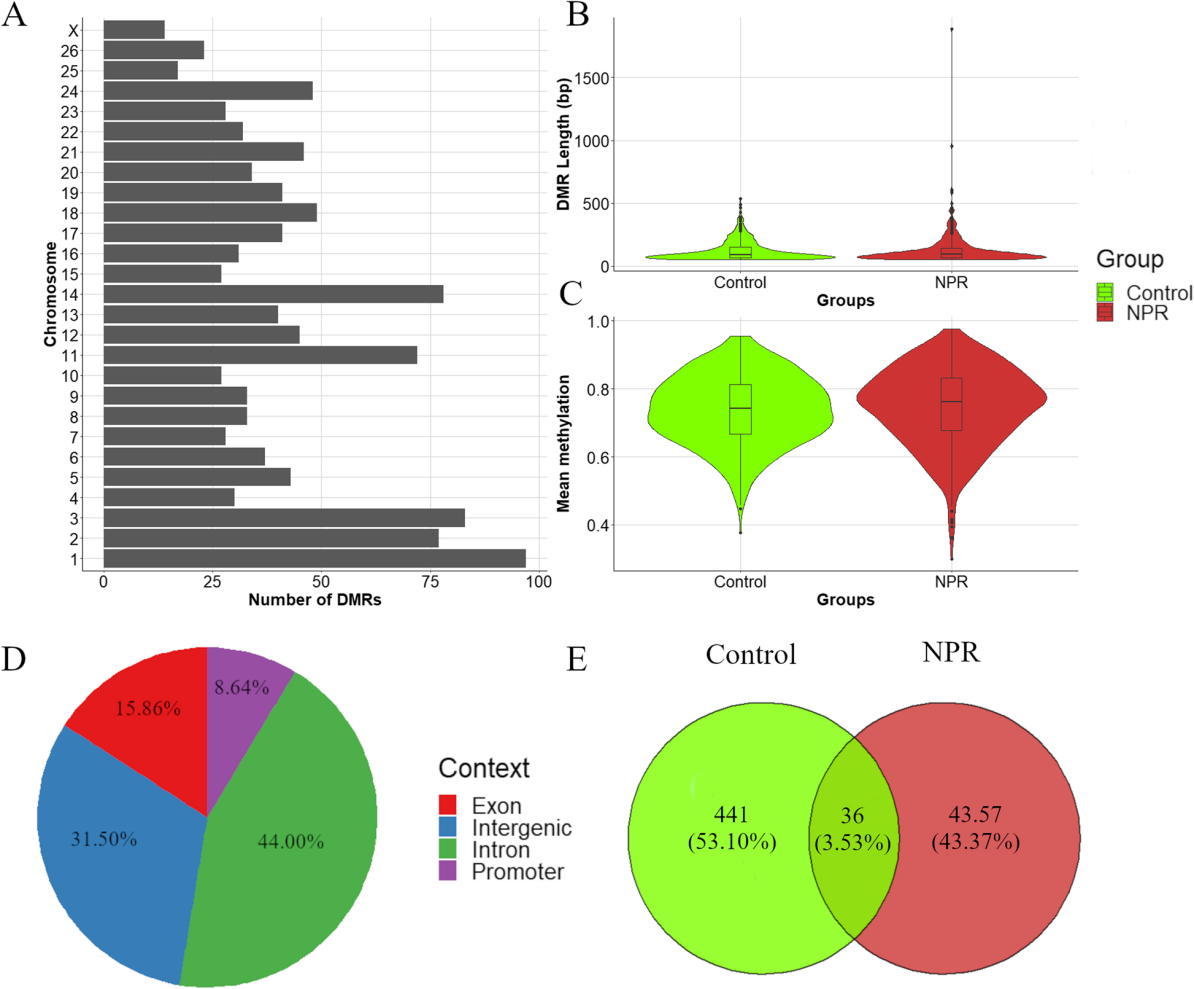
Distribution of differentially methylated regions (DMRs) per chromosome (**A**), per length in base pairs, bp (**B**) and by mean methylation (**C**). The percentage of DMRs per each gene context (promoter in purple, exon in red, intron in green, and intergenic in blue) is shown in the pie plot (**D**). The number of genes shared between the DMRs hypermethylated for the nutritional protein restriction (NPR) or control group is shown in the Venn Diagram (**E**). For the violin plots and Venn Diagram, the mean methylation level for DMRs hypermethylated in the control group is displayed in green, and the mean methylation for those DMRs hypermethylated in the NPR group is indicated in red

In total, 32,247 DMLs were identified between the NPR and C groups. A circular Manhattan plot showing the distribution of the adjusted p values of the DMLs and a density plot showing the distribution of the significant DMLs across the genome is presented in Fig. 1. The most prominent association peaks were observed on chromosomes 1, 7, 11, 20, and 24. The calling of DMRs among these DMLs resulted in the identification of 1,154 DMRs (Supplementary Table 3). The distribution of these DMRs across chromosomes, their lengths and the corresponding mean methylation distribution are shown in Fig. 2A and B. The largest number of DMRs was observed on chromosome 1 (97 DMRs), and the smallest number of DMRs was identified on chromosome X (14 DMRs). The comparison of the lengths and mean methylation levels of the DMRs did not show substantial general differences between the NPR and C groups. Additionally, it is important to highlight that the mean methylation level was compared with the methylation level of hypermethylated DMRs in the NPR and C groups (Fig. 2C). Consequently, despite the general similarity between the distribution of the mean methylation level, all of these DMRs presented differential methylation patterns between the NPR and C groups. The percentage of DMRs annotated in each gene context (promoter, exon, intron, and intergenic) and the number of genes assigned exclusively or simultaneously to DMRs that were hypermethylated in the NPR or C group are shown in Fig. 2 D and E, respectively. The majority of DMRs were mapped to introns or intergenic regions, with promoters and exons corresponding to 8.64% and 15.86% of the DMR genomic context, respectively. Only 36 genes were simultaneously assigned to DMRs that were hypermethylated in the NPR and C groups. In total, 540 genes were exclusively assigned to DMRs that were hypermethylated in the NPR group, while 441 genes were exclusively assigned to DMRs that were hypermethylated in the C group.

### Candidate genes and functions associated with DMRs identified between NPR and control ewes

**Fig. 3:**
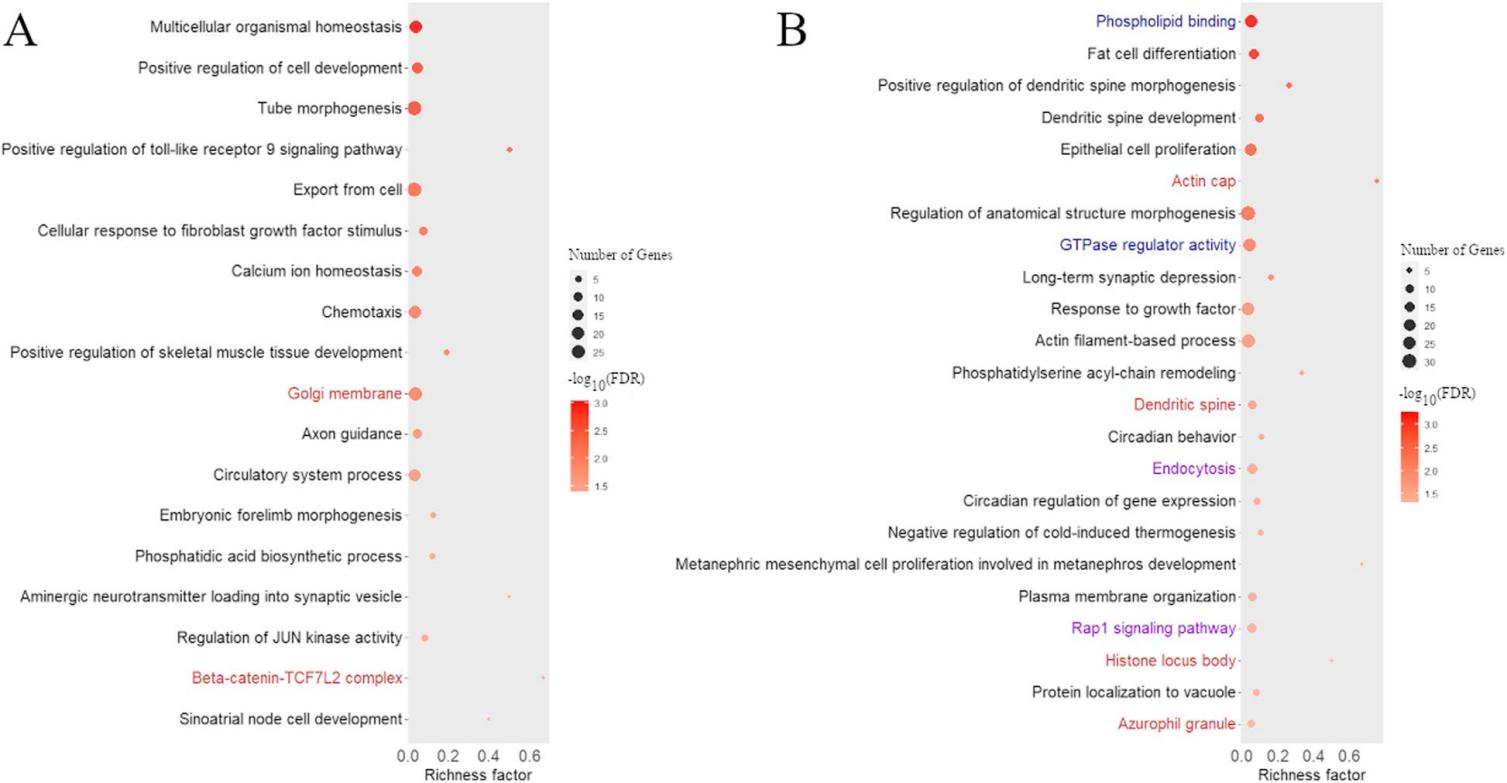
Bubble plots displaying the enrichment results for Gene Ontology (GO) terms (Biological Processes in black, Molecular Functions in blue, and Cellular Components in red) and metabolic pathways (in purple) obtained for the genes harboring hypermethylated DMRs in the control group (**A**) and nutritional protein restriction group (**B**). The area of the circles in the plot corresponds to the number of associated genes for that term, while the shade of red represents the adjusted *P* value (the darker the red shade, the smaller the *P* value is). The x-axis shows the richness factor, which corresponds to the ratio between the number of associated genes for a specific and the total number of genes assigned for this trait in the database

The ten DMRs with the largest absolute AreaStat (the sum of the test statistics of all CpG sites within the DMR) are shown in Table 1. The regions harboring the most prominent peaks of DMLs (located on chromosomes 1, 11, 20, and 7) were scrutinized to identify the DMRs and the candidate genes in those regions. These regions were identified by a constant pattern of decreasing p values and false discovery rates (FDRs) < 1 × 10^–100^. Figure 3 shows the genomic context of the two regions harboring the largest number of DMRs on chromosomes 1 (5 DMRs in a 45.5 Kb interval from 112,851,283–112,896,784) and 20 (6 DMRs in a 1,155.25 Kb interval from 50,206,967–51,362,216). All DMRs in the abovementioned region of chromosome 1 were hypermethylated in the NPR group (Supplementary Fig. 2A). On the other hand, among the 6 DMRs called in the analyzed region of chromosome 20, four were hypermethylated, and 2 were hypomethylated in the NPR group (Supplementary Fig. 2B). The DMRs mapped on chromosome 1 are located in a region composed of several tRNAs and two LOCs (mRNA-basic proline-rich protein-like and mRNA-collagen alpha-1(I) chain-like). Additionally, a second region on chromosome 1 harbored one of the DMRs with the ten highest AreaStat values. The DMR mapped in this region (1:3,049,876–3050313) was also hypermethylated in the NPR group and mapped to exonic/intronic regions of *PER2*. From the DMRs mapped on chromosome 20, the two hypomethylated DMRs in the NPR group were assigned to the *GMDS* gene. Regarding the hypermethylated DMRs in the NPR group mapped on chromosome 20, two were assigned to the *FOXC1* gene, one was assigned to the *LOC114109593* gene, and one was assigned to the *LOC121817372* gene. Additionally, two DMRs were called in the analyzed region of chromosome 7, one hypomethylated in the NPR group and one hypermethylated in the NPR group. Both DMRs were mapped to intronic regions of the *TTC7B* gene. On chromosome 11, only one DMR was called in the regions harboring prominent peaks of DMLs. This DMR was mapped to 11:42,240,935–42,241,421, which corresponds to the exonic region of the *CAVIN1* gene. This DMR was hypomethylated in the NPR group (mean methylation = 0.518) compared to the C group (0.754).

**Table 1:** Differentially methylated regions (DMRs) with the ten highest absolute AreaStat (sum of the test statistics of all CpG sites within the DMR) identified in the comparison between the nutritional protein restriction (NPR) and control group

Coordinate	Length (bp)	Number mCG	Mean methylation NPR	Mean methylation Control	AreaStat	Gene	Gene context
1:112,885,455–112,887,339	1885	397	0.362	0.191	6875.091	*TRNAL-CAG_5*	Promoter/Exon
20:50,694,985–50695939	955	204	0.531	0.306	3474.655	*FOXC1*	Intergenic
1:112,896,353–112,896,784	432	122	0.299	0.157	2061.900	*TRNAG-UCC_11*	Promoter/Exon
1:112,851,283–112,851,733	451	116	0.441	0.184	1883.910	*TRNAG-GCC_2*	Promoter/Exon
11:42,240,935–42241421	487	95	0.518	0.754	-1516.839	*CAVIN1*	Exon
1:3,049,876–3050313	438	49	0.880	0.436	704.617	*PER2*	Exon/Intron
1:112,869,229–112,869,556	328	57	0.544	0.193	644.766	*TRNAL-CAG_3*	Promoter
7:100,234,881–100235250	370	40	0.375	0.821	-612.966	*LOC121820014*	Intron
12:79,002,203–79002581	379	52	0.588	0.220	596.674	*KIF21B*	Intergenic
8:74,623,072–74623442	371	52	0.416	0.209	516.377	*LOC114116137*	Intergenic

In total, 40 Gene Ontology (GO) terms and 2 KEGG pathways were enriched (FDR < 0.05) in genes assigned to the identified DMRs. One GO term was shared between the genes harboring DMRs that were hypermethylated or hypomethylated in the NPR group (ameboidal-type cell migration). The genes assigned to DMRs that were hypomethylated in the NPR group were associated with 20 enriched GO terms (16 biological processes and four cellular component terms). For the genes assigned to DMRs that were hypermethylated in the NPR group, 19 GO terms (15 biological processes, two molecular functions and two cellular components) and two KEGG pathways were identified as enriched. All enriched terms and pathways are shown in Fig. 3, and the complete enrichment results are available in Supplementary Table 4. In general, the enriched terms for the genes harboring DMRs that were hypomethylated in the NPR group (hypermethylated in the C group) were related to the regulation of organismal development, morphogenesis, and homeostasis. On the other hand, the DMRs that were hypermethylated in the NPR group were associated with more specialized enriched terms, such as phospholipid binding, fat cell differentiation, epithelial cell proliferation, circadian behavior, circadian regulation of gene expression, and negative regulation of cold-induced thermogenesis.

**Fig. 4:**
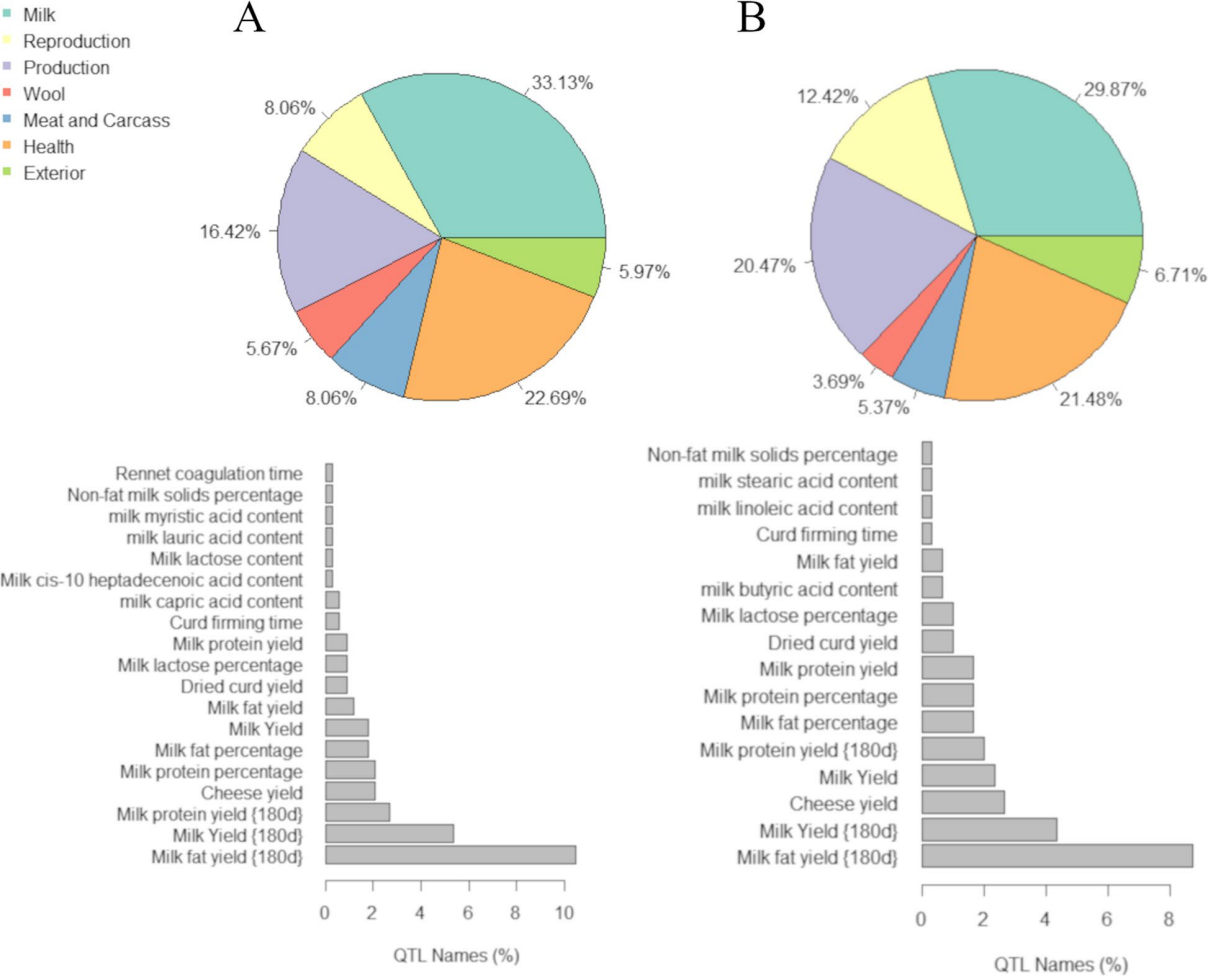
Percentages of quantitative trait loci (QTL) category (upper part) annotated for the differentially methylated regions in a 250 Kb interval (downstream and upstream) hypermethylated in NPR (**A**) and control groups (**B**). The bar plots (lower part) show the percentage of each milk-related trait assigned to the milk QTL category

The milk-related quantitative trait loci (QTL) were the most frequent QTL class annotated within the coordinates (± 250 kb) of the DMRs that were hypermethylated in the NPR (33.13%), and C (29.87%) groups (Fig. 4). Enrichment analysis suggested that the trait assigned to each QTL class was enriched within the DMR coordinates evaluated (data not shown). For both NPR and C hypermethylated DMRs, the most frequent traits assigned to the annotated milk-related QTLs were milk fat yield {180d}, milk yield {180d}, milk protein yield {180d}, and cheese yield. A total of 68 genes were identified harboring DMRs mapped within the coordinates of milk-related QTLs (Supplementary Table 5).

### Correlations between milk production and differentially methylated regions

The mean values of FP, PP, DE, SCC, and MY for the 28 ewes evaluated in the current study are available in Supplementary Table 6. In comparisons between the NPR and C groups, one animal was excluded since it was the only ewe with a parity of four lambs and because this ewe showed substantially larger numbers of SCC across all 11 time points sampled. The comparison of the log-transformed values between the NPR and C groups indicated no effect of the dietary group on FP, PP, DE, SCC, or MY (Table 2).

**Table 2:** Mean (± standard deviation) values for fat, protein and dry extract percentages and number of somatic cells counts in the milk between the nutritional protein restriction (NPR) and control (C) groups

Milk production trait	Group		*p*-value*
	NPR	Control	
Fat (%)	5.23 (± 0.57)	5.60 (± 0.34)	0.054
Protein (%)	4.79 (± 0.26)	4.86 (± 0.27)	0.438
Dry extract (%)	15.85 (± 0.79)	16.25 (± 0.51)	0.109
Somatic cell count (× 10^3^ cells/ml)	119.61 (± 57.42)	200.44 (± 315.64)	0.526
Milk yield (Kg)	2.80 (± 0.57)	2.96 (± 0.46)	0.431

^*^P-value computed for the log-transformed values

**Fig. 5:**
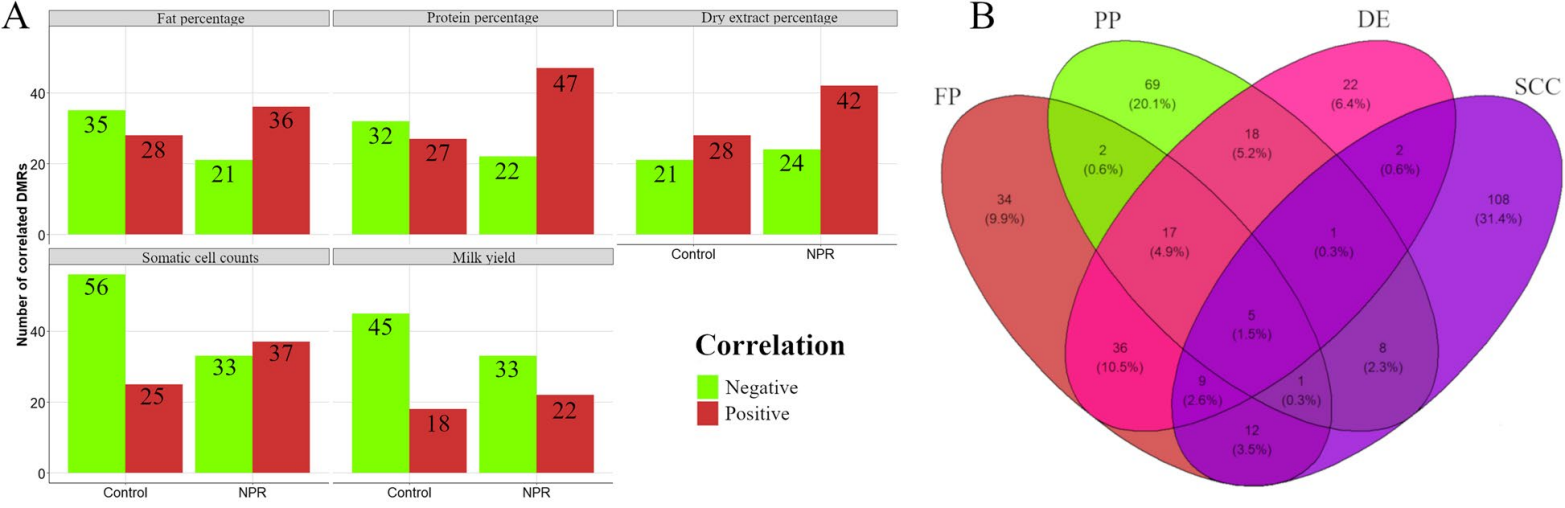
Functional characterization of differentially methylated regions (DMR) significantly correlated with fat (FP), protein (PP), dry extract (DE) percentages, somatic cell counts (SCC), and milk yield (MY). **A** Number of DMRs positively (in red) and negatively (in green) correlated with each evaluated trait identified individually using the data comprising the nutritional protein restriction (NPR) and control group. **B** Venn diagram showing the number of genes harboring significantly correlated DMRs, shared between FP (in red), PP (in green), DE (in pink), and SCC (in purple). MY was not included in the Venn diagram because just one enriched GO term was identified for the genes harboring significantly correlated DMRs with MY

**Fig. 6:**
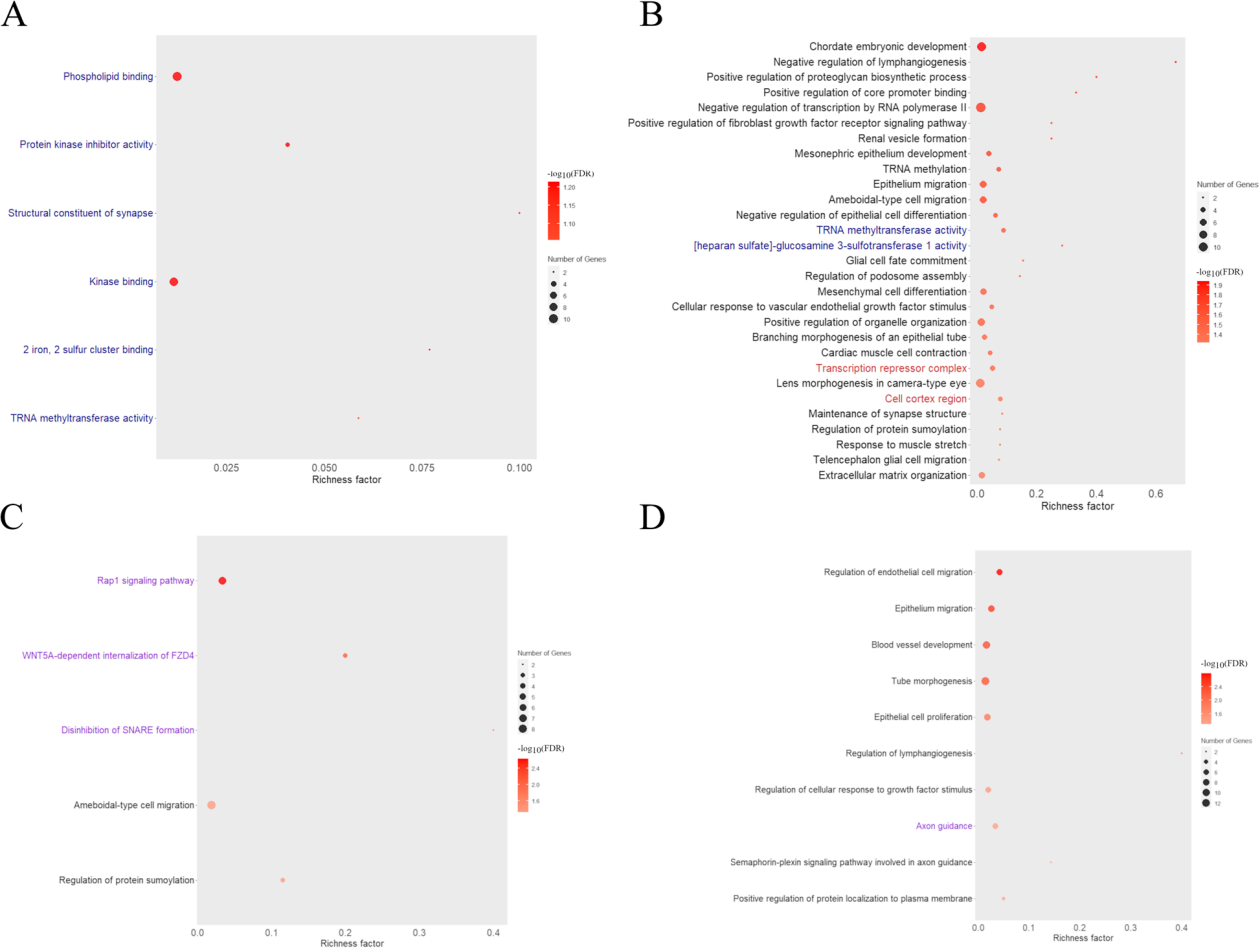
Bubble plots displaying the enrichment results for Gene Ontology (GO) terms (Biological Processes in black, Molecular Functions in blue, and Cellular Components in red) and metabolic pathways (in purple) obtained for the genes harboring significantly correlated DMRs with FP (**A**), PP (**B**), DE (**C**), and SCC (**D**). The area of the circles in the plot corresponds to the number of associated genes for that term, while the shade of red represents the adjusted p-value (the darker the red shade, the smallest the *p*-value). The x-axis shows the richness factor, which corresponds to the ratio between the number of associated genes for a specific and the total number of genes assigned for this trait in the database

In total, 120, 128, 115, 151, and 118 DMRs were significantly correlated with FP, PP, DE, SCC and MY, respectively (Fig. 5A and Supplementary Table 7). The number of genes harboring DMRs correlated with different milk-related traits is shown in Fig. 5B. SCC was the trait with the largest number of unique genes harboring significantly correlated DMRs (108 genes). Five genes harbored DMRs that were correlated with all four milk traits: *EPHA2*, *SHANK2*, *LOC114117507*, *LOC101121820*, and *LOC114110015*. The enriched GO terms and/or metabolic pathways of the genes harboring DMRs that were significantly correlated with each trait individually are shown in Fig. 6 A-D, respectively. The complete list of enriched GO terms and metabolic pathways identified for the genes harboring DMRs correlated with FP, PP, DE, and SCC is available in Supplementary Table 8. There were no enriched GO terms or metabolic pathways among the genes harboring DMRs that were significantly correlated with FP based on the 5% FDR threshold. Therefore, in Fig. 6A, a 10% FDR threshold was adopted for identifying enriched terms. Additionally, just one GO term, “Ruffle membrane” (from the cellular component class), was identified as enriched for the genes positively correlated with MY even applying a FDR 10% threshold. The GO terms associated with the genes harboring DMRs that were significantly correlated with FP were related to phospholipid binding (among other processes). For the genes harboring DMRs correlated significanty with PP, important GO terms were observed, such as Cellular response to vascular endothelial growth factor stimulus, Response to muscle stretch, and Regulation of protein sumoylation. Regarding the GO terms enriched for the genes harboring DMR correlated with SCC, several terms associated with cellular proliferation and migration were observed (Regulation of endothelial cell migration, Epithelium migration, Epithelial cell proliferation, Regulation of cellular response to growth factor stimulus). Table 3 shows 48 DMRs that were significantly correlated with at least one of the milk traits (FP, PP, DE, and SCC) and were mapped within the genic regions (promoter, exon, and intron) of 44 genes associated with at least one of the enriched GO terms or metabolic pathways. Among these 48 DMRs, 18 significant correlations (11 negative and 7 positive) were observed for 17 DMRs mapped within 17 genes for FP. For PP, 18 significant correlations (5 negative and 13 positive) were observed for 18 DMRs mapped within 17 genes. The 14 significant correlations observed with DE (2 negatives and 12 positives) corresponded to 14 DMRs mapped within 13 genes. For SCC, 18 significant correlations (10 negatives and 8 positives) were observed, which were mapped within the genic regions of 17 genes harboring 17 DMRs associated with at least one of the enriched GO terms or metabolic pathways. Finally, for MY three significant correlations were observed between DMRs mapped in three different genes: *FAM107A* (0.543), *PSD2* (-0.674), and *TESC2* (-0.648).

**Table 3:** Differentially methylated regions (DMRs) identified in the comparison between the nutritional protein restriction (NPR) and control group mapped within gene coordinates (promoter, exon, and introns) identified in the comparison between NPR and control group and significantly correlated with fat (FP), protein (PP), dry extract (DE) percentages, somatic cells count (SCC), and milk yield (MY)

Gene	Trait	r^2^	p-value	Group	Coordinate	Length (bp)	nCG	Mean methylation NPR	Mean methylation Control	Gene region	Associated enriched term
*ANXA11*	DE	-0.684	0.009	NPR	25:34,323,916–34,323,982	67	9	0.713	0.517	Intron	Phospholipid binding
*ANXA11*	FP	0.670	0.011	C	25:34,323,916–34,323,982	67	9	0.713	0.517	Intron	
*ANXA11*	FP	-0.587	0.030	NPR	25:34,323,916–34,323,982	67	9	0.713	0.517	Intron	
*AP2A2*	DE	0.670	0.011	C	21:46,837,762–46,837,854	93	6	0.292	0.955	Intron	WNT5A-dependent internalization of FZD4
*AP2A2*	PP	0.644	0.015	C	21:46,837,762–46,837,854	93	6	0.292	0.955	Intron	
*ASAP1*	PP	-0.710	0.006	C	9:23,458,839–23,459,051	213	25	0.537	0.212	Intron	Regulation of podosome assembly, Positive regulation of organelle organization
*CCBE1*	SCC	0.538	0.050	C	23:58,879,912–58,879,995	84	11	0.412	0.641	Intron	Regulation of endothelial cell migration, Epithelium migration, Blood vessel development, Tube morphogenesis, Regulation of lymphangiogenesis, Regulation of cellular response to growth factor stimulus
*CDKN1C*	FP	-0.596	0.028	NPR	21:45,168,358–45,168,504	147	13	0.460	0.767	Intron	Protein kinase inhibitor activity, Epithelial cell proliferation, Regulation of cellular response to growth factor stimulus
*CDKN1C*	SCC	0.613	0.022	NPR	21:45,168,358–45,168,504	147	13	0.460	0.767	Intron	
*CHD8*	PP	-0.618	0.021	C	7:24,079,637–24079718	82	4	0.714	0.919	Exon/Intron	Chordate embryonic development, Negative regulation of transcription by RNA polymerase II
*CHD8*	SCC	0.560	0.040	C	7:24,079,637–24079718	82	4	0.714	0.919	Exon/Intron	
*COL5A1*	SCC	-0.648	0.015	C	3:1,633,932–1,634,020	89	7	0.235	0.902	Intron	Blood vessel development
*CTBP2*	DE	0.609	0.024	C	22:44,176,758–44,176,942	185	7	0.735	0.421	Intron	Kinase binding, Structural constituent of synapse, Negative regulation of transcription by RNA polymerase II, Transcription repressor complex, Cell cortex region, Maintenance of synapse structure
*CTBP2*	DE	0.552	0.044	C	22:44,085,601–44085731	131	11	0.386	0.715	Intron	
*CTBP2*	FP	0.692	0.008	C	22:44,085,601–44085731	131	11	0.386	0.715	Intron	
*CTBP2*	PP	0.547	0.046	C	22:44,114,597–44,114,726	130	10	0.443	0.760	Intron	
*CTBP2*	PP	-0.609	0.021	NPR	22:44,193,718–44,193,809	92	9	0.772	0.504	Intron	
*CTNNB1*	DE	0.648	0.015	NPR	19:13,887,469–13,887,576	108	4	0.354	0.691	Intron	Chordate embryonic development, Positive regulation of proteoglycan biosynthetic process, Positive regulation of core promoter binding, Negative regulation of transcription by RNA polymerase II, Mesonephric epithelium development, Renal vesicle formation, Positive regulation of fibroblast growth factor receptor signaling pathway, Negative regulation of epithelial cell differentiation, Glial cell fate commitment, Mesenchymal cell differentiation, Positive regulation of organelle organization, Branching morphogenesis of an epithelial tube, Transcription repressor complex, Lens morphogenesis in camera-type eye, Cell cortex region, Response to muscle stretch, Regulation of protein sumoylation, Telencephalon glial cell migration, Rap1 signaling pathway, Regulation of protein sumoylation
*CTNNB1*	PP	0.662	0.012	NPR	19:13,887,469–13,887,576	108	4	0.354	0.691	Intron	
*DGKD*	FP	-0.560	0.040	C	1:7,607,203–7607313	111	12	0.784	0.461	Promoter/Intron	Kinase binding, Phospholipid binding
*EGR3*	PP	0.622	0.020	C	2:42,913,872–42,913,944	73	4	0.308	0.658	Intron	Ameboidal-type cell migration, Epithelium migration, Cellular response to vascular endothelial growth factor stimulus
*EPHB2*	SCC	-0.536	0.048	C	2:243,811,728–243,811,961	234	10	0.662	0.380	Intron	Blood vessel development, Tube morphogenesis, Axon guidance, Positive regulation of protein localization to plasma membrane
*ESYT2*	DE	0.824	0.000	NPR	4:121,076,547–121076925	379	26	0.484	0.868	Intron	Phospholipid binding
*ESYT2*	FP	0.791	0.001	NPR	4:121,076,547–121076925	379	26	0.484	0.868	Intron	
*EVL*	DE	0.622	0.020	C	18:62,590,356–62590448	93	10	0.664	0.417	Promoter	Ameboidal-type cell migration, Epithelium migration, Positive regulation of organelle organization, Rap1 signaling pathway, Ameboidal-type cell migration, Epithelium migration
*EVL*	PP	0.544	0.044	NPR	18:62,713,451–62,713,511	61	11	0.835	0.494	Intron	
*EVL*	SCC	-0.568	0.034	C	18:62,713,451–62,713,511	61	11	0.835	0.494	Intron	
*FAM107A*	MY	0.543	0.047	NPR	19:42,907,370–42907424	55	6	0.700	0.476	Intron	Ruffle membrane
*GATA3*	SCC	-0.662	0.012	C	13:12,393,887–12,393,956	70	9	0.337	0.560	Exon	Regulation of endothelial cell migration, Epithelium migration, Tube morphogenesis, Epithelial cell proliferation, Regulation of cellular response to growth factor stimulus
*GFI1*	PP	0.594	0.025	C	1:69,739,282–69,739,347	66	12	0.458	0.257	Promoter	Negative regulation of transcription by RNA polymerase II, Transcription repressor complex
*GSN*	PP	-0.591	0.029	NPR	2:2,230,782–2230864	83	5	0.717	0.395	Intron	Regulation of podosome assembly, Positive regulation of organelle organization, Cardiac muscle cell contraction, Response to muscle stretch
*HMGB1*	SCC	0.569	0.037	NPR	10:30,611,368–30611452	85	7	0.434	0.639	Intron	Regulation of endothelial cell migration, Epithelium migration, Epithelial cell proliferation
*ITGB2*	FP	0.603	0.022	C	1:265,503,556–265503666	111	18	0.591	0.292	Intron	Kinase binding
*KRAS*	SCC	0.559	0.038	NPR	3:190,372,975–190373031	57	6	0.466	0.685	Promoter	Tube morphogenesis, Axon guidance
*MCF2L*	FP	0.560	0.037	NPR	10:85,791,273–85,791,355	83	8	0.351	0.620	Intron	Phospholipid binding
*NEDD4L*	PP	0.538	0.050	C	23:57,726,528–57,726,758	231	26	0.868	0.533	Exon/Intron	Cardiac muscle cell contraction, Lens morphogenesis in camera-type eye
*NKX6-3*	PP	0.657	0.013	C	26:35,908,505–35908812	308	21	0.838	0.423	Exon	Negative regulation of transcription by RNA polymerase II, Negative regulation of epithelial cell differentiation
*NLRP6*	FP	0.547	0.046	NPR	21:47,417,071–47417281	211	22	0.449	0.747	Promoter/Intron	Phospholipid binding
*PDPK1*	SCC	-0.552	0.044	C	24:2,553,687–2,553,863	177	19	0.813	0.430	Intron	Regulation of endothelial cell migration, Epithelium migration, Blood vessel development, Tube morphogenesis, Epithelial cell proliferation, Regulation of cellular response to growth factor stimulus, Axon guidance, Positive regulation of protein localization to plasma membrane
*PLXNA4*	SCC	-0.538	0.050	NPR	4:97,917,124–97,917,301	178	15	0.341	0.616	Intron	Axon guidance, Semaphorin-plexin signaling pathway involved in axon guidance
*PRKCA*	DE	0.565	0.038	NPR	11:61,970,930–61971146	217	32	0.867	0.640	Intron	Rap1 signaling pathway, WNT5A-dependent internalization of FZD4, Disinhibition of SNARE formation, Ameboidal-type cell migration
*PRKCA*	FP	0.538	0.050	NPR	11:61,970,930–61971146	217	32	0.867	0.640	Intron	
*PRKCG*	DE	-0.569	0.037	NPR	14:62,283,367–62,283,417	51	5	0.643	0.933	Exon/Intron	Rap1 signaling pathway, WNT5A-dependent internalization of FZD4, Disinhibition of SNARE formation
*PSD2*	MY	-0.675	0.010	C	5:48,628,000–48628177	178	6	0.907	0.376	Promoter/Intron	Ruffle membrane
*RNH1*	SCC	0.574	0.032	C	21:47,184,999–47,185,053	55	10	0.332	0.706	Exon	Blood vessel development, Tube morphogenesis
*RPL7L1*	PP	0.552	0.044	NPR	20:16,546,702–16546774	73	7	0.403	0.598	Exon	Chordate embryonic development
*SASH1*	SCC	0.604	0.025	NPR	8:72,918,784–72,918,860	77	7	0.298	0.537	Promoter/Exon/ Intron	Regulation of endothelial cell migration, Epithelium migration, Blood vessel development, Tube morphogenesis
*SCARB2*	FP	-0.559	0.038	C	6:91,833,512–91,833,633	122	7	0.553	0.282	Intron	Phospholipid binding
*SESTD1*	FP	-0.551	0.041	C	2:130,911,122–130911184	63	6	0.627	0.357	Intron	Phospholipid binding
*SHANK2*	DE	0.640	0.016	NPR	21:44,415,614–44,415,779	166	10	0.661	0.365	Intron	Structural constituent of synapse, Maintenance of synapse structure
*SHANK2*	FP	0.547	0.046	NPR	21:44,415,614–44,415,779	166	10	0.661	0.365	Intron	
*SHANK2*	PP	0.569	0.037	NPR	21:44,415,614–44,415,779	166	10	0.661	0.365	Intron	
*SHANK2*	SCC	-0.591	0.029	C	21:44,415,614–44,415,779	166	10	0.661	0.365	Intron	
*SYDE1*	DE	0.613	0.022	C	5:7,861,395–7,861,467	73	8	0.707	0.490	Exon	Ameboidal-type cell migration
*SYNE3*	PP	-0.647	0.012	NPR	18:58,083,719–58083776	58	5	0.556	0.348	Intron	Lens morphogenesis in camera-type eye
*TESC*	My	-0.650	0.015	C	17:57,987,904–57987979	76	5	0.405	0.714	Intron	Ruffle membrane
*TOM1L2*	DE	0.592	0.026	C	11:34,328,017–34328106	90	19	0.454	0.706	Intron	Kinase binding, Phospholipid binding
*TOM1L2*	FP	0.770	0.001	C	11:34,328,017–34328106	90	19	0.454	0.706	Intron	
*TRIML1*	FP	-0.596	0.028	C	26:16,845,521–16,845,593	73	11	0.842	0.633	Exon/Intron	Kinase binding
*TRMT44*	PP	0.556	0.039	NPR	6:115,436,923–115,437,013	91	5	0.737	0.276	Promoter/Intron	tRNA methylation, TRNA methyltransferase activity
*XDH*	FP	-0.563	0.036	C	3:92,263,184–92,263,240	57	5	0.382	0.679	Intron	2 iron, 2 sulfur cluster binding, Blood vessel development, Tube morphogenesis, Epithelial cell proliferation, Regulation of cellular response to growth factor stimulus
*XDH*	SCC	0.669	0.009	NPR	3:92,263,184–92,263,240	57	5	0.382	0.679	Intron	
*XDH*	SCC	-0.569	0.034	C	3:92,263,184–92,263,240	57	5	0.382	0.679	Intron	
*ZFP36*	SCC	-0.591	0.029	NPR	14:48,460,374–48460444	71	4	0.496	0.192	Exon	Epithelial cell proliferation
*ZMIZ1*	DE	0.701	0.007	NPR	25:34,086,596–34086829	234	6	0.414	0.815	Intron	Chordate embryonic development, Regulation of protein sumoylation, Telencephalon glial cell migration, Regulation of protein sumoylation
*ZMIZ1*	FP	0.714	0.006	NPR	25:34,086,596–34086829	234	6	0.414	0.815	Intron	
*ZMIZ1*	PP	0.688	0.008	NPR	25:34,086,596–34086829	234	6	0.414	0.815	Intron	
*ZNF423*	SCC	-0.763	0.002	NPR	14:17,670,197–17670272	76	8	0.813	0.445	Intron	Regulation of cellular response to growth factor stimulus
*ZNF536*	DE	0.560	0.040	NPR	14:40,460,902–40461150	249	14	0.803	0.521	Exon	Negative regulation of transcription by RNA polymerase II
*ZNF536*	FP	0.705	0.006	NPR	14:40,460,902–40461150	249	14	0.803	0.521	Exon	
*ZNF536*	PP	0.543	0.048	NPR	14:40,460,902–40461150	249	14	0.803	0.521	Exon	

*Bp* Base pair, *nCG* Number of methylated cytosines within the DMR

In general, DMRs that were hypermethylated in the NPR group were associated with enriched terms involved in biological processes that are functionally relevant to mammary gland production, such as phospholipid binding, fat cell differentiation, epithelial cell proliferation, the response to growth factors, and the regulation of circadian behavior. On the other hand, the hypermethylated DMRs in the C group (or hypomethylated DMRs in the NPR group) were associated with enriched GO terms related to organism homeostasis, the regulation of cell development, export from the cell, tissue development and synapsis-related processes. These processes may be related to the general homeostasis, development and activity of the mammary gland.

It is important to highlight that the number of significant correlations presented is larger than the number of DMRs because each DMR was tested for correlation with each of the considered milk production traits, and these correlations were also tested separately in the NPR and C groups.

## Discussion

Epigenetic marks are relevant functional targets of nutritional stress conditions with important effects on the metabolic status of the organism [33–35]. Nutritional status is an important factor affecting milk production and quality in dairy sheep, in which fat-related characteristics tend to be more easily affected than milk protein levels [36]. Indeed, the supplementation of protein and amino acids only marginally affects milk protein levels [36]. Recently, our research group reported the absence of an effect of NPR on milk production traits such as SCC during the first lactation of Assaf ewes [16]. This analysis was performed in the same dataset of animals evaluated in the current study, where the absence of an effect of NPR in the prepubertal stage on FP, PP, DE, and SCC was also observed during the second lactation. Despite the absence of an effect on milk-related production traits, a relatively large number of DMRs (1154) were observed between the milk somatic cells of animals subjected to NPR or not. An important aspect to consider is that the substitution of soybean meal from the diet implies a modification in the qualitative composition of the NPR diet beyond the reduction of crude protein (42.3% in NPR animals). Therefore, observed effects on DNA methylation might not be solely attributed to protein restriction but also to (potential) differences in metabolizable energy intake and/or modified intake of other nutrients such as ADF, ADL or lipids.

On chromosome 1, two regions showed a pattern of increased significance for DMLs. Indeed, the DMRs mapped within these regions were among the top 10 DMRs with the highest absolute AreaStat values. The first region comprised 5 DMRs in a 45.5 Kb interval from 112,851,283–112,896,784, and it was mapped within a region with several annotated tRNAs. In lactating rats, perinatal protein restriction affects the free amino acid and fatty acid profile of the milk [37]. Pulina et al. (2006) [36] described divergent effects of diets with different protein contents on milk composition. The authors suggested that these contrasting results might be related to the milk production level achieved in those animals. The epigenetic regulation of genomic tRNA loci is described in the literature as an important mechanism regulating a plethora of biological processes, including metabolic regulation and age-related adaptations, and patterns of differential methylation in regions with clusters of several tRNAs have previously been described [38–41]. The other region on chromosome 1 harbored one DMR (1:3,049,876–3050313), which was mapped within exonic and intronic regions (depending on the transcript) of the *PER2* gene. This DMR was more than twice as hypermethylated in the NPR group as in the C group. The *PER2* gene is associated with the control of circadian rhythm in the central nervous system and peripheral organs [42]. An important function of *PER2* is the regulation of *PPARγ*, a nuclear receptor that plays crucial roles in adipogenesis, the inflammatory response and insulin sensitivity [43–45]. In mice, *PER2* deficiency results in drastic reductions in total triacylglycerol and nonesterified fatty acid levels in white adipocytes [46]. In sheep, the dietetic suppression of melatonin alongside constant exposure to light increases basal lipolysis and induces the overexpression of *PER2* and *PPARγ*, among other adipogenic/thermogenic and circadian clock genes [47]. In addition, in cattle, the silencing of *PER2* results in the suppression of lipid synthesis in the mammary gland through the regulation of *SREBF1* and *PPARγ* [48]. The results obtained in the comparison between NPR and C animals for FP suggest potential differences between the groups that could not be observed here due to sample size limitations. The *PER2* region is hypomethylated in the NPR animals in the current experiment, and it might be acting in the inhibition of its expression in the mammary gland of these animals, consequently affecting the FP in the milk samples. Among other functional candidate genes harboring DMRs in the most prominent regions analyzed in the current study, *FOXC1* and *CAVIN1* stand out. A DMR mapped in an intergenic region close to *FOXC1* (20:50,694,985–50,695,939) was hypermethylated in the NPR group. In mice, the overexpression of *FOXC1* results in the suppression of lobuloalveologenesis and lactation associated with higher percentages of estrogen receptor-, progesterone receptor-, or ki67-positive mammary epithelial cells during the lactation stage [49]. The epigenetic control of *FOXC1* during mammary gland development was previously described in humans [50]. In the current study, one of the exons of the *CAVIN1* gene was found to harbor a DMR (11:42,240,935–42,241,421) that was hypermethylated in the C group compared with the NPR group. This gene encodes Caveolae Associated Protein 1, which acts alongside caveolin-1 to create a unique lipid environment in caveolae [51]. Caveolae are domains localized on the cell surface in vertebrates that play important roles in cell migration and mechanoprotection [52, 53]. The activity and function of caveolin-1 are modulated by *CAVIN1* in different processes, as observed for the oncogenic activity of caveolin-1 [54]. In addition, caveolin-1 is downregulated during lactation through the action of prolactin, and caveolin-1-deficient mice show accelerated mammary gland development and premature lactation [55, 56]. The same authors demonstrated that the recombinant overexpression of caveolin in HC11 cells could inhibit the prolactin-induced activation of β-casein promoter activity and synthesis [55]. Therefore, *FOXC1* and *CAVIN1* have the potential to emerge as functional candidate genes involved in the epigenetic control of mammary gland production in sheep.

The analysis of QTLs previously described in the regions harboring the DMRs in the NPR and C groups demonstrated that most annotated QTLs were related to milk traits. Among all milk-related QTLs, the regions harboring DMRs in the current study were more frequently associated with DE yield (protein and fat, mainly), MY and cheese yield. These results suggest a potential role of these DMRs in regulating biological processes associated with milk-related production traits.

To identify DMRs resulting from nutrient protein restriction with the potential to regulate milk-related production traits, we tested the correlations between the mean methylation levels within the 1154 DMRs and FP, PP, DE, SCC, and MY within the NPR and C groups individually. In total, DMRs mapped within 424 genes were significantly correlated with at least one of the milk traits evaluated. The phospholipid-binding molecular function was the most enriched GO term associated with the genes harboring DMRs correlated with FP. Phospholipids are crucial molecules that depend on coordinated flux and availability for the proper secretion of milk fat and other components [57]. The genes harboring DMRs correlated with PP resulted in the largest number of enriched GO terms. Among these terms, the large number of terms associated with the development of different structures, such as the differentiation of mesenchymal cells and epithelium migration, can be highlighted. The interactions between mesenchymal and epithelial cells are crucial for the proper development of the mammary gland [58]. The Rap1 metabolic signaling pathway was the most enriched for those genes harboring DMRs correlated with DE. Recently, Rap1 signaling was identified as being enriched in coexpressed gene networks from bovine mammary epithelial cells contrasted by high- and low-fat rates [59]. Additionally, the Rap1 signaling pathway is an important component of the proliferation and differentiation of mammary epithelial cells with the potential to increase milk production [60–62]. The number of enriched terms linked to endothelial and epithelial cell proliferation and migration associated with the genes harboring DMRs correlated with SCC stands out. The epithelial cells that are shed into milk during lactation have cellular characteristics of terminally differentiated luminal cells, and the analysis of immunohistochemical markers for proliferation indicated an increase in epithelial cell proliferation from early lactation to late lactation in cows [63]. This finding is corroborated by the intense renewal of epithelial cells during lactation, where most mammary cells present in the mammary gland at the end of lactation are formed after calving in dairy cows [64]. The involvement of the genes harboring DMRs correlated with milk production traits in biological processes crucial for mammary gland development and production reinforces the possibility that these epigenetic markers act in a compensatory mechanism related to the protein restriction to which the animals were subjected. Only five genes harbored DMRs correlated with all four milk traits, among which three were uncharacterized loci: *LOC114117507*, *LOC101121820*, and *LOC114110015*, while the other two genes were *EPHA2* and *SHANK2*. *SHANK2* encodes one of the major scaffold proteins of excitatory synapses [65]. The establishment of epithelial cell polarity is controlled by the formation of tight junctions with the participation of the Rap1 signaling pathway through the binding of *SHANK2* to atypical protein kinase C [66]. The DMR mapped in the intronic region of *SHANK2* (21:44,415,614–44,415,779) was hypermethylated under NPR and was positively correlated with FP, PP, and DE, while it was negatively correlated with SCC. *EPHA2* has been demonstrated to act as an important component of mammary gland development, with roles in epithelial proliferation and branching morphogenesis, a process that is more active during puberty [67]. A DMR mapped to an intergenic region close to *EPHA2* (2:249,637,801–249,638,050) was hypermethylated in the NPR group compared with the C group and showed negative correlations with FP, PP, and DE. In contrast, a positive correlation was observed with SCC. The highest absolute correlations were observed between the DMR (4:121,076,547–121,076,925) mapped to the intronic region of the *ESYT2* gene and DE and FP. *ESYT2* encodes Extended Synaptotagmin 2, a member of the Synaptotagmin complex, which acts together with SNARE (Soluble N-Ethylmaleimide-Sensitive Factor Attachment Protein Receptor) proteins as a mediator of the specific fusion of transport vesicles and exocytosis with potential functions in mammary epithelial cells [68]. Additionally, *ESYT2* controls the dynamics of Ca^2+^ in different types of cells [69], and it is suggested to play a direct role in lipid transport [70]. The DMR mapped in the intronic region of *ESYT2* (4:121,076,547–121,076,925) was hypermethylated in the C group compared with the NPR group, and it was positively correlated with DE and FP.

Among the other genes harboring DMRs that were significantly correlated with milk traits, the functional impact of *GATA3*, *HMGB1*, *CTBP2*, and *TOM1L2* can be highlighted. *GATA3* encodes a transcription factor expressed in luminal breast epithelial cells that regulates cell proliferation in this tissue [71]. In the current study, a DMR in *GATA3* (13:12,393,887–12,393,956) was hypermethylated in the C group and negatively associated with SCC according to the values from the animals of the C group. In mice, changes in the expression of *HMGB1,* encoding the high mobility group box 1 protein, are observed during mammary gland development, with lower values occurring during lactation and involution [72]. Here, a hypermethylated DMR in the C group (10:30,611,368–30611452), mapped within *HMGB1*, was positively correlated with SCC. *CTBP2* genes have previously been linked with transcriptional corepressor activity in the liver, acting as metabolic sensors responsible for regulating glucose and lipid homeostasis [73]. Three different DMRs in *CTBP2* were positively correlated with DE (22:44,176,758–44,176,942 and 22:44,085,601–44,085,731), FP (22:44,085,601–44,085,731) and PP (22:44,114,597–44,114,726). All of these positive correlations were observed in relation to the values obtained for these traits in the C group samples. Additionally, one DMR in *CTBP2* (22:44,193,718–44,193,809) was negatively correlated with the PP values of the samples in the NPR group. *TOM1L2* encodes a protein involved in membrane trafficking and endocytosis [74, 75]. In goats, hypomethylated DMR on *TOM1L2* was previously identified by comparing mammary gland samples from dry and lactation periods [76]. Here, a DMR in the intronic region of *TOM1L2* (11:34,328,017–34,328,106), which was hypermethylated in the C group, was positively correlated with DE and FP. We also highlight that *SREBF1* is a major regulator of lipid metabolism in the mammary gland, and a putative regulatory effect of this DMR on *SREBF1* cannot be disregarded [77–79]. Despite the absence of functionally relevant enriched terms for the DMRs correlated with MY, it is interesting to highlight an intronic DMR (9:21,489,061.21489139) mapped in the Thyroglobulin (*TG*) gene which was negatively correlated with FP (-0.538) and positively correlated with MY (0.622). Polymorphims in the *TG* were previously associated with 305-day milk fat percent and otal lactation fat percentage in dairy cows and buffalo [80, 81].

The results obtained here pinpoint putative functional candidate genes for milk production traits in sheep based on differential methylation patterns. Future studies can leverage the results obtained here to better evaluate the regulatory elements within the DMRs described in the current study. The analysis of active promoters and enhancers for these functional candidate genes has the potential to better characterize the regulatory mechanisms enrolled with the expression of those genes and the phenotypic variance observed among individuals [82]. Additionally, analysis comprising the methylation level of the candidate DMRs reported here for each cellular type available in milk samples might help to understand the contribution of each cell type to production traits. It is important to mention that the methylation status can be a dynamic process and change across time and development. For example, in sheep, the analysis of differential methylation profiles between fetal and adult muscular tissue was useful in providing new insights regarding the development of these tissues and the identification of potential functional candidate genes that might be associated with meat quality and production-related traits [83]. Therefore, a longitudinal analysis of DMRs in the milk somatic cells across lactations would help to identify time-specific methylation marks and candidate genes associated with milk components and productions in sheep. Despite the absence of significant differences between the NPR and C groups for the milk-related traits evaluated here, it is important to mention that these animals showed a significant difference in the body weight at the end of the nutritional protein restriction trial as shown in Pelayo et al. (2023) [84]. The data evaluated here is composed by a limited set of phenotypes at the second lactation of these animals. Therefore, a longitudinal study evaluating the association of the DMRs described here with the same phenotypes (and additional milk-related traits) is crucial to better understand the functionality of these methylation marks.

The mammary gland is a complex organ that provides a combination of immune and epithelial cells. The milk somatic cells are mainly cells from the immune system, such as lymphocytes, macrophages, and polynuclear cells. The reasons for using milk somatic cells in the current studies can be classified into practical, evidence-based, and animal welfare. The animal welfare aspect is related to the global demands for animal research, which are based, among other aspects, on the 3Rs (Replacement, Reduction, and Refinement) principle. These principles are considered a systematic approach to animal experimentation that puts the well-being of the animals front and center. Based on the 3Rs recommendations, an invasive approach (mammary-gland biopsy) should be replaced when an efficient proxy is available (Milk somatic cells analysis). The evaluation of mammary epithelial cells requires the application of invasive methodologies, such as mammary biopsies. From the practical point of view, mammary biopsies are not viable in commercial flocks, which is the case of the animals used in the current study, due to potential mammary tissue damage and disruptions in the lactation process. One of the main goals of the current study is to identify potential biomarkers that could be evaluated in commercial herds. Therefore, the use of milk somatic cells is a viable alternative for the evaluation of such biomarkers. Indeed, from an evidence-based point of view, despite the majority of the milk somatic cells being immune cells, in goats and sheep, several studies described an association between milk somatic cells and different milk production traits, such as milk yield, protein, mineral, and fat contents, and cheese making [85]. The use of WGBS for the investigation of genetic mechanisms associated with milk production is a relatively new approach in all livestock species, especially in sheep. Therefore, there are not studies showing the actual correlation between epigenetic markers in milk somatic cells and mammary gland tissues. However, together with the correlations between milk somatic cells and milk production traits described above, some additional information reinforces the viability of using milk somatic cells for the functional evaluation of the mammary gland. For example, studies in other species showed that using the transcriptome from milk somatic cells is a good representation of the mammary gland transcriptome obtained from biopsies [86–89]. Furthermore, in a study conducted in our research group [90], we proved that transcriptome analysis of somatic cells in milk from healthy animals is an excellent proxy to analyze specific functional changes in mammary gland epithelial cells (changes in the expression profile of genes related to the de novo synthesis of milk fat and protein) in response to specific feeding strategies or challenges such as milk fat depression. Additionally, the evaluation of specific biomarkers for mammary gland activity, such as the stearoyl‐CoA desaturase (SCD1) expression, suggested milk somatic cells of lactating cows as an indicator of SCD1 activity within the mammary gland [90]. This conclusion was reached due to the high correlation between the expression values of SCD1 between the tissues and also because the SCD1 expression in milk somatic cells was significantly related to Δ9-desaturase indices in milk, which are commonly used as an indicator of SCD1 activity within the mammary gland [87]. Despite the absence of a study evaluating the different methylation levels between the different cell types present in the milk somatic cells, some interesting comparisons can be made from results obtained from human blood samples. Similarly to the milk somatic cells, whole blood samples are also composed by different cellular types (in different proportions). Talens et al. (2010) [91] analyzed the variations of DNA methylation in different human tissues, with an exciting focus on the correlations between the methylation profile of different whole blood cellular types. The results obtained suggested that the majority of variation observed in the methylation pattern was not explained by the cellular heterogeneity, and when some variation in DNA methylation that could be explained by variation in cellular heterogeneity was observed, this variation was generally small, and associations were of borderline significance. Therefore, based on the abovementioned evidences, we consider the use of milk somatic cells an interesting alternative for the epigenetic evaluation of the sheep mammary gland.

## Conclusion

The results presented here suggest a relevant effect of DMRs on the expression of genes associated with milk production and lipid metabolism in sheep. The DMRs were identified using a strict statistical threshold, and the experimental groups were distinguished from each other by protein restriction in the diet during the prepubertal stage. Although it is impossible to disregard effects on other milk characteristics, such as fatty acid and free amino acid profiles, there were no longer-term effects (on the second lactation) of protein restriction on FP, PP, DE, and SCC. Consequently, the abovementioned results might suggest a potential impact of the DMRs mapped within these candidate genes responsible for regulating key components of mammary gland development and production. It is important to highlight that the absence of a significant correlation of some of the DMRs with the evaluated milk traits did not exclude the possibility of a functional impact of these epigenetic markers in the mammary gland. In light of the above, the results obtained from the current study help to better understand the epigenetic mechanisms associated with milk production in the mammary gland of dairy sheep. The functionality of these epigenetic markers could be validated in further studies applying techniques such as ATAC-seq and the identification of expression QTLs (eQTLs) associated with the DMRs identified herein and other milk-related production traits.

### Supplementary Information

**Additional file 1.**

## Data Availability

All sequence data obtained in the current study were uploaded to the European Nucleotide Archive under the accession number PRJEB56589 (https://www.ebi.ac.uk/ena/browser/view/PRJEB56589).

## Abbreviations

C: Control group
DE: Dry extract percentage
DML: Differentially methylated loci
DMR: Differentially methylated regions
FDR: False-discovery rate
FP: Fat percentage
NPR: Nutritional protein restriction group
PP: Protein percentage
QTL: Quantitative trait loci
SCC: Somatic cell counts
WGBS: Whole genome bisulfite sequencing
