# Supplementary material for: Epigenetic regulation of functional candidate genes for milk production traits in dairy sheep subjected to protein restriction in the prepubertal stage
Source: BMC Genomics. 2023 Sep 1;24:511. doi: 10.1186/s12864-023-09611-y (PMC10472666; doi:10.1186/s12864-023-09611-y)
Supplement: Supplementary file 1 — Additional file 1. [file 12864_2023_9611_MOESM1_ESM.zip › Suppl_Material/SupplementaryFigure1.pdf]

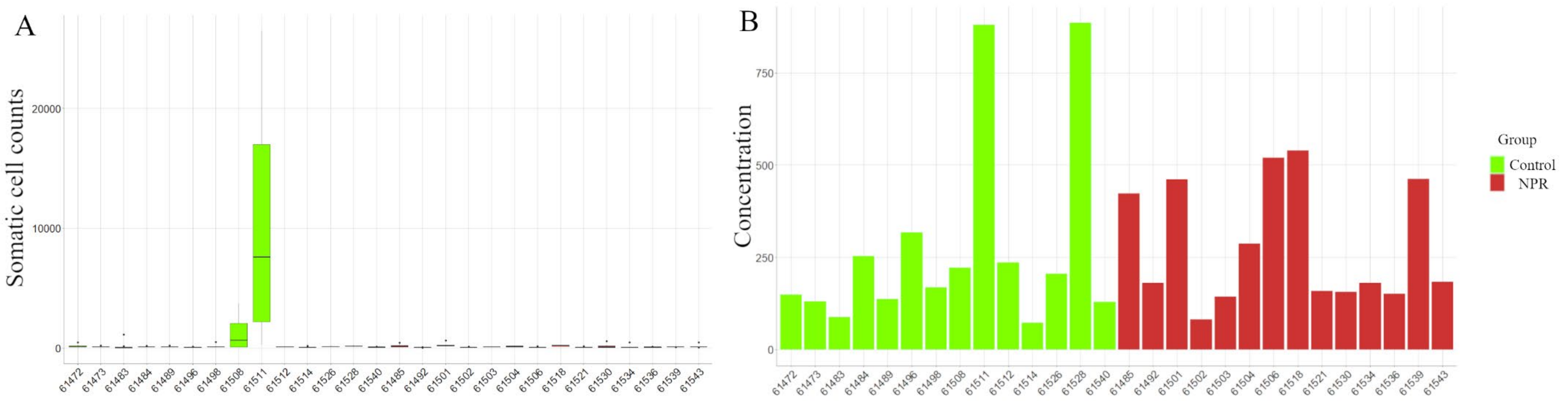

Supplementary Figure 1: A) Distribution of somatic cell counts (x10^3 cells/ml) per samples. B) Concentration of DNA (ng/mL) obtained from the 100 mL of milk sampled from each ewe. In green and red are shown the control and nutritional protein restriction (NPR) samples, respectively.
