# Supplementary material for: Epigenetic regulation of functional candidate genes for milk production traits in dairy sheep subjected to protein restriction in the prepubertal stage
Source: BMC Genomics. 2023 Sep 1;24:511. doi: 10.1186/s12864-023-09611-y (PMC10472666; doi:10.1186/s12864-023-09611-y)
Supplement: Supplementary file 1 — Additional file 1. [file 12864_2023_9611_MOESM1_ESM.zip › Suppl_Material/SupplementaryTable1.pdf]

Supplementary Table 1. Ingredients and chemical composition of the diet provided to the two groups of ewes during the nutritional protein restriction challenge.

**Diet Composition**<br>Ingredients (g/kg of fresh matter)	**C**	**NPR**	Change in NPR (g/kg fresh matter)
Maize grain	400	503	103
Barley grain	300	377	77
Soybean meal 47	180	----	-180
Wheat bran	60	60	---
Lard	10	10	---
Molasses (beet)	20	20	---
Minerals and vitamins	30	30	---
**Chemical composition**	C	NPR	Change in NPR (%)
Dry matter (DM; g/kg)	869	870	+0.11
Ash (g/kg DM)	61.0	51.8	-15.1
Crude protein (g/kg DM)	182	105	-42.3
Neutral detergent fibre (g/kg DM)	119	121	+1.68
Acid detergent fibre (g/kg DM)	46.5	39.1	-15.9
Acid detergent lignin (g/kg DM)	6.6	8.5	+28.8
Ether extract (g/kg DM)	33.8	43.8	+29.6
Gross energy (kcal/kg DM)	4396	4346	+0.01

Control group (C), and nutritional protein restriction group (NPR)
