# Supplementary material for: Epigenetic regulation of functional candidate genes for milk production traits in dairy sheep subjected to protein restriction in the prepubertal stage
Source: BMC Genomics. 2023 Sep 1;24:511. doi: 10.1186/s12864-023-09611-y (PMC10472666; doi:10.1186/s12864-023-09611-y)
Supplement: Supplementary file 1 — Additional file 1. [file 12864_2023_9611_MOESM1_ESM.zip › Suppl_Material/SupplementaryTable2.pdf]

Supplementary Table 2: Mapping statistics for the whole genome bisulfite sequencing (WGBS) data from the milk of the Assaf ewes included in the present study.

Sample Alias	ID	Group	Mapping	Mapped pairs	Unmapped pairs	%mCG	%mCHG	%mCHH
Dmilk1_D	61485	NPR	0.597	151033960	101766683	71.615	1.376	1.436
Dmilk10D	61530	NPR	0.699	184055695	79085652	70.099	1.315	1.398
Dmilk11D	61534	NPR	0.694	188205272	82848171	71.016	1.355	1.449
Dmilk12D	61536	NPR	0.690	177161263	79535167	70.456	1.328	1.42
Dmilk13D	61539	NPR	0.691	179838249	80553174	70.408	1.325	1.433
Dmilk14D	61543	NPR	0.690	210266057	94368786	70.738	1.327	1.413
Dmilk2_D	61492	NPR	0.635	166458488	95497213	71.458	1.338	1.39
Dmilk3_D	61501	NPR	0.677	184971568	88266726	72.53	1.368	1.437
Dmilk4_D	61502	NPR	0.635	188795299	108292520	71.358	1.362	1.44
Dmilk5_D	61503	NPR	0.688	189990114	85996589	71.241	1.351	1.456
Dmilk6_D	61504	NPR	0.688	203337526	92034600	70.857	1.378	1.457
Dmilk7_D	61506	NPR	0.691	214062888	95680540	73.736	1.363	1.421
Dmilk8D	61518	NPR	0.679	191976593	90870657	73.326	1.328	1.412
Dmilk9D	61521	NPR	0.680	200385640	94259250	69.884	1.346	1.428
Dmilk1_C	61472	Control	0.630	190522769	111969311	71.675	1.359	1.411
Dmilk10C	61512	Control	0.690	188323588	84534374	72.408	1.333	1.409
Dmilk11C	61514	Control	0.678	176176511	83649814	71.913	1.304	1.402
Dmilk12C	61526	Control	0.692	207873883	92307717	71.31	1.34	1.421
Dmilk13C	61528	Control	0.689	188251798	85008597	72.186	1.366	1.464
Dmilk14C	61540	Control	0.686	241948807	110607430	70.406	1.364	1.43
Dmilk2_C	61473	Control	0.623	198400668	119859842	71.859	1.358	1.39
Dmilk3_C	61483	Control	0.615	162319506	101438670	71.635	1.333	1.394
Dmilk4_C	61484	Control	0.609	157900861	101331364	70.667	1.333	1.387
Dmilk5_C	61489	Control	0.623	187233217	113382175	70.931	1.342	1.405
Dmilk6_C	61496	Control	0.623	160610468	97155766	71.959	1.319	1.395
Dmilk7_C	61498	Control	0.624	162024368	97484806	69.966	1.338	1.394

Dmilk8_C	61508	Control	0.690	235459604	105842325	71.086	1.362	1.443
Dmilk9_C	61511	Control	0.683	187713977	87239334	73.916	1.334	1.407

NPR: Nutritional protein restriction; %mCG: percentage of methylated cytosine followed by guanine; %mCHG: percentage of methylated cytosine followed by H (where H equal to C, T or A) and a guanine; %mCHH: percentage of methylated cytosine followed by two H (where, H equal to C, T or A).
