# Supplementary material for: Epigenetic regulation of functional candidate genes for milk production traits in dairy sheep subjected to protein restriction in the prepubertal stage
Source: BMC Genomics. 2023 Sep 1;24:511. doi: 10.1186/s12864-023-09611-y (PMC10472666; doi:10.1186/s12864-023-09611-y)
Supplement: Supplementary file 1 — Additional file 1. [file SupplementaryFigure2.pdf › Suppl_Material/SupplementaryFigure2.pdf]

A

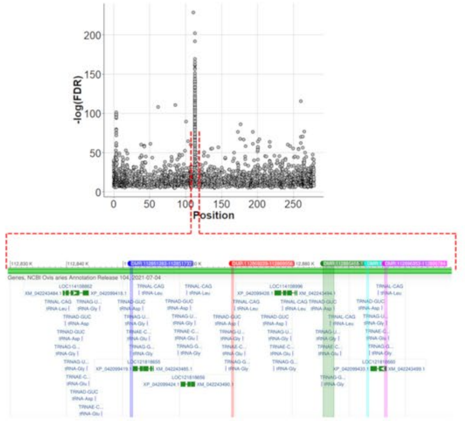

DMR	Coordinate	Length (bp)	Number of mCG	Mean methylation NPR	Mean Methylation control	Gene symbol	Gene region
	1:112851283-112851733	451	116	0.441	0.184	*TRNAG-UCC_11*	Promoter/Exon
	1:112869229-112869556	328	57	0.544	0.193	*TRNAL-CAG_3*	Promoter
	1:112885455-112887339	1885	397	0.362	0.191	*TRNAL-CAG_5*	Promoter/Exon
	1:112893163-112893512	350	38	0.409	0.195	*TRNAG-GCC_7*	Promoter
	1:112896353-112896784	432	122	0.299	0.157	*TRNAG-UCC_11*	Promoter/Exon

B

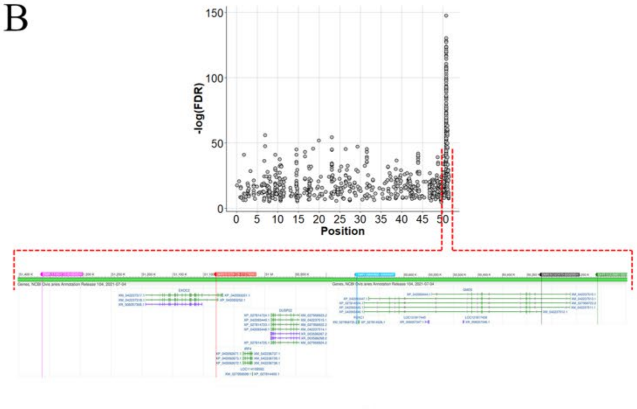

DMR	Coordinate	Length (bp)	Number of mCG	Mean methylation NPR	Mean Methylation control	Gene symbol	Gene region
	20:50206967-50207135	169	22	0.413	0.655	*GMDS*	Intergenic
	20:50320879-50320934	56	5	0.474	0.785	*GMDS*	Promoter/Intron
	20:50694833-50694907	75	17	0.602	0.387	*FOXC1*	Intergenic
	20:50694985-50695939	955	204	0.531	0.306	*FOXC1*	Intergenic
	20:51079128-51079391	264	28	0.866	0.656	*LOC114109593*	Intron
	20:51362111-51362216	106	10	0.701	0.300	*LOC121817372*	Intergenic

Supplementary Figure 2: Genomic context for the differentially methylated regions (DMRs) on chromosome 1 (5 DMRs in a 45.5 Kb interval from 112,851,283-112,896,784) (A) and 20 (6 DMRs in a 1,155.25 Kb interval from 50,206,967-51,362,216) (B) mapped in regions where differentially methylated loci (DMLs) reached a threshold of False-Discovery Rate $<10^{-100}$.
